# EEG correlates of social interaction at distance

**DOI:** 10.12688/f1000research.6755.3

**Published:** 2016-01-14

**Authors:** William Giroldini, Luciano Pederzoli, Marco Bilucaglia, Patrizio Caini, Alessandro Ferrini, Simone Melloni, Elena Prati, Patrizio Tressoldi

**Affiliations:** 1 Evanlab, Firenze, 50023, Italy; 2 Dipartimento di Psicologia Generale, Università di Padova, Padova, 35131, Italy

**Keywords:** mental entanglement, EEG, ERP, bootstrap, Monte Carlo.

## Abstract

This study investigated EEG correlates of social interaction at distance between twenty-five pairs of participants who were not connected by any traditional channels of communication.

Each session involved the application of 128 stimulations separated by intervals of random duration ranging from 4 to 6 seconds. One of the pair received a one-second stimulation from a light signal produced by an arrangement of red LEDs, and a simultaneous 500 Hz sinusoidal audio signal of the same length. The other member of the pair sat in an isolated sound-proof room, such that any sensory interaction between the pair was impossible.

An analysis of the Event-Related Potentials associated with sensory stimulation using traditional averaging methods showed a distinct peak at approximately 300 ms, but only in the EEG activity of subjects who were directly stimulated. However, when a new algorithm was applied to the EEG activity based on the correlation between signals from all active electrodes, a weak but robust response was also detected in the EEG activity of the passive member of the pair, particularly within 9 – 10 Hz in the Alpha range. Using the Bootstrap method and the Monte Carlo emulation, this signal was found to be statistically significant.

## Introduction

The study of EEG correlates of social interaction is a hot topic in the world of social neuroscience, described as follows by Cacioppo & Berntson (2002): “ *Social neuroscience addresses fundamental questions about the mind and its dynamic interactions with the biological systems of the brain and the social world in which it resides”*.

The study of EEG correlates of social interaction ranges from simple face-to-face motor interactions (e.g. Hari *et al.*, 2013), to empathy ( Singer & Lamm, 2009), to interpersonal motor co-ordination ( Oullier *et al.*, 2008; Sebanz & Knoblich, 2009). A recent review of the current status of the field, particularly in reference to social cognition, is given by Chatel-Goldman *et al.* (2013). This review highlights the importance of not underestimating possible non-local mechanisms that can emerge from person-to-person interactions. These mechanisms are defined as “ *dependent operations between two or more brains that operate at least in part on shared information content*” and are also described as *interactive alignment*, *resonance, phase synchronization, and non-local correlations*.

Is it conceivable that these mechanisms can be detected even when two persons are mentally interacting without the possibility of sensory information exchange?

This possibility is rarely studied, not so much because of technical or methodological difficulties, but because the prevailing view is that the human mind can only receive information through the five senses and anything else is impossible. Nonetheless, if we assume that the human mind is also capable of receiving and processing information transmitted from other than the five senses, it becomes possible to investigate the characteristics of mental activity related to the interaction between two sensorily isolated individuals.

This model of mind function, also defined as “non-local” because it is not limited to the spatial and temporal confines of the five senses, is predicted by various theoretical models. For example, according to Dual-Aspect Monism ( Atmanspacher, 2012), there is neither a material reality nor a mental reality – rather, they are two different aspects of one reality. These mental characteristics are also consistent with Generalized Quantum Theory (GQT) proposed by Walach & von Stillfried (2011), and Filk & Römer (2011).

This theory predicts mind-to-mind and mind-to-matter non-local correlations similar to the entanglement phenomena observed in quantum physics if the following conditions are fulfilled:

*1) A system is given, inside which subsystems can be identified. Entanglement phenomena will be best visible if the subsystems are sufficiently separated such that local observables pertaining to different subsystems are compatible*.

*2) There is a global observable of the total system, which is complementary to local observables of the subsystems*.

*3) The total system is in an entangled state. For instance, eigenstates of the global observable are typically entangled states*.

The theory of Generalized Entanglement assumes that a distant social interaction between two persons who know each other must satisfy these requirements:

a) the two persons represent two subsystems of a single larger one created by their relationship, and
b) this relationship constitutes an entangled state, and furthermore that
c) the measurable psychological and physiological variables represent the system’s comprehensive characteristic even though measured individually.

However it is important to point out that when dealing with mental observables, the identification and operationalization of the subsystems within a global one, and their complementary and/or compatible characteristics, is still an open problem.

The study presented here is a further addition to this field of research. In comparison to other research, our specific objectives are:

1) To determine the difference in power or other statistical characteristics of the EEG signal between the person receiving the physical stimulus and his/her mentally connected partner;
2) Determine the latency period, if any, between the EEG signals and the stimulus of both partners;
3) Determine the frequency ranges of EEG activity that best represent the connection between the subject pair.

To date, we have identified 29 published studies starting from 1965 which have addressed this possibility using EEG activity as a dependent variable (see Supplementary Material). Unfortunately due to the types of EEG sources analyzed and the statistical analyses used to test the existence of a non-local social interaction, it is very difficult to meta-analyze them. Our study is a further contribution to this line of research, but for the first time within the social neuroscience and the GQT framework. Furthermore we will present a new method for the analysis of EEG signals which proved superior to the classical ones.

## Methods

### Subjects

Six Italian Caucasian healthy adults were chosen for the experiment, comprised of five men and one woman, with an average age of 35.5 years (standard deviation = 8.3).

They were selected among the members of the EvanLab, the private laboratory involved in this study. The criteria for their voluntary inclusion were their mutual friendship (> 10 years), and their experience in being able to maintain prolonged focused concentration – a product of their familiarity with meditation and other practices requiring control of mental activities.

### Statement of Ethics

The use of experimental subjects is in accordance with ethical guidelines as outlined in the Declaration of Helsinki, and the study has been approved by the Ethical Committee of the University of Padova’s Department of General Psychology. Before taking part in the experiment, each subject gave his/her informed consent in writing after having read a description of said experiment.

### Equipment

A software program, available at http://dx.doi.org/10.6084/m9.figshare.1466876, especially written by one of our co-authors (GW) administered the sequence of stimuli and synchronized the EEG recordings from each member of the pairs. EEG activity was measured using two Emotiv ^®^ EEG Neuroheadsets, equipped with 14 EEG channels, connected via WiFi to a Windows PC.

The technical details are: 14 electroencephalography channels based on international location from 10 to 20 (AF3, F7, F3, FC5, T7, P7, O1, O2, P8, T8, FC6, F4, F8, AF4, plus two reference electrodes). The mastoid electrodes (M1, M2) served as reference points against which the voltage generated from all other electrodes was compared. The sample frequency of the Emotiv ^®^ headsets is 128 Hz, with a bandwidth from 0.2 to 45 Hz, with a built-in fifth order low-pass digital filter as well as two notch filters at 50 and 60 Hz respectively as protection against noise produced by the local electricity network. The Emotiv ^®^ EEG has a proprietary wireless network connection at a frequency of 2.4 GHz.

### Stimuli

The auditory stimulus was composed of a 500 Hz sinusoid applied through 32 Ohm Parrot ZIK ^®^ earphones at a volume of about 80 dB. The visual stimulation was from high intensity red LEDs in a 4×4 arrangement placed approximately one meter from the subject being stimulated. The subject kept his/her eyes closed because the light could easily be detected through the eyelids.

### Procedure

The members of each pair were placed in two separate rooms approximately five meters from each other. Each room was sound- and light-proof, so as to block out any and all external sensory information.

Between these two rooms was a control room with two computers connected to the Emotiv ^®^ headsets and from which the research assistant controlled the sensory stimulation program and each partner’s EEG recording (see Figure S1 in Supplementary Material). The software program in use ensured that the signals coming from the two EEG headsets were recorded simultaneously (to within 8 ms).

The partner designated as “Sender” was given the following instructions: *“When you are ready, relax and be prepared to receive a visual and auditory stimulus which you will send to your partner. To assist your mental connection with him/her, concentrate on his/her photo before starting the experiment. Your only task is to mentally transmit what you will perceive, while limiting your body movements to prevent interference with your EEG activity. You will perceive 128 stimulations of 1 second each, separated by pauses of random length lasting 4 to 6 seconds in order to avoid predictable rhythms. The experiment will last about 10 minutes.”*

The partner designated “Receiver” was given the following instructions: “ *When you are ready, relax and be prepared to receive the stimuli sent from your partner. To assist your mental connection with him/her, you will see a facial photo of him/her before starting the experiment. Your task is to mentally connect with him/her and try to perceive the stimulus he/she is receiving, while keeping your body still to prevent interference with your EEG activity. The experiment will last about 10 minutes.”*

Once the quality of the EEG signals was confirmed, and with the consent of the subjects, the research assistant began running the experiment’s program. To prevent either subject from predicting when the first stimulus would be given, it was preceded by a period of silence of random duration from 2 to 3 minutes.

At the end of the experiment, after a period of rest, in most cases (if subjects agreed and time allowed) the role of each subject was reversed.

All together data from 25 pairs of subjects was collected over three days. The raw data are available at http://dx.doi.org/10.6084/m9.figshare.1466876 which include details of pairings.

### Bias control

To avoid any experimenter’s effect, the research assistant who managed the software for the data acquisition were blind to the exact start of the stimulation sequence, given the randomization of the duration of the first pre-stimulation period as described above.

The reduction of the risk of any conventional communication between the pair of participants, was guaranteed by the sensory isolation of the two rooms were they were placed as already described. The only remaining possibility was to speak aloud each other, but this event could clearly be noticed by the research assistant.

## Results

### Data analysis

**Figure 1.**
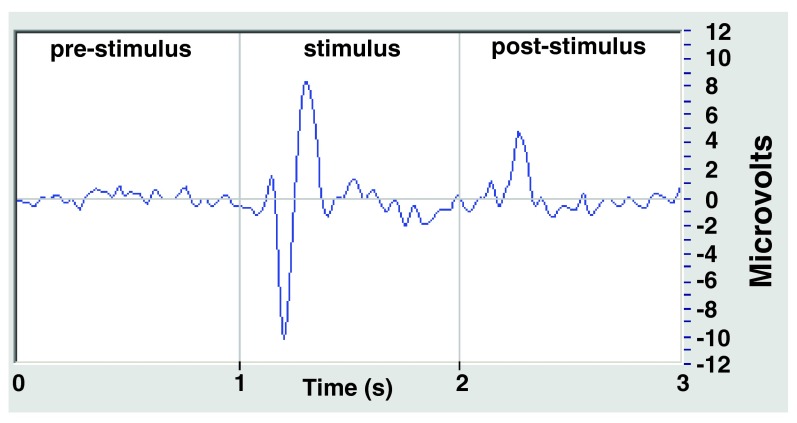
Typical example of an evoked potential obtained from processing a Sender’s signals. The graph is an average of 128 stimuli and 14 EEG channels. Usually two peaks are seen, a negative and a positive one, about 250 to 300 ms after the stimulus begins, and a minor peak at about 250 ms after the stimulus ceases.

Collection of the evoked potential was initially conducted by filtering the signals in the 1–12 Hz band followed by normalization (see software code of Appendix 1 at http://dx.doi.org/10.1101/022046), hence employing the traditional averaging method of time- and phase-locked epochs. The typical result of an evoked potential obtained from Senders can be seen in the graph in Figure 1.

**Figure 2.**
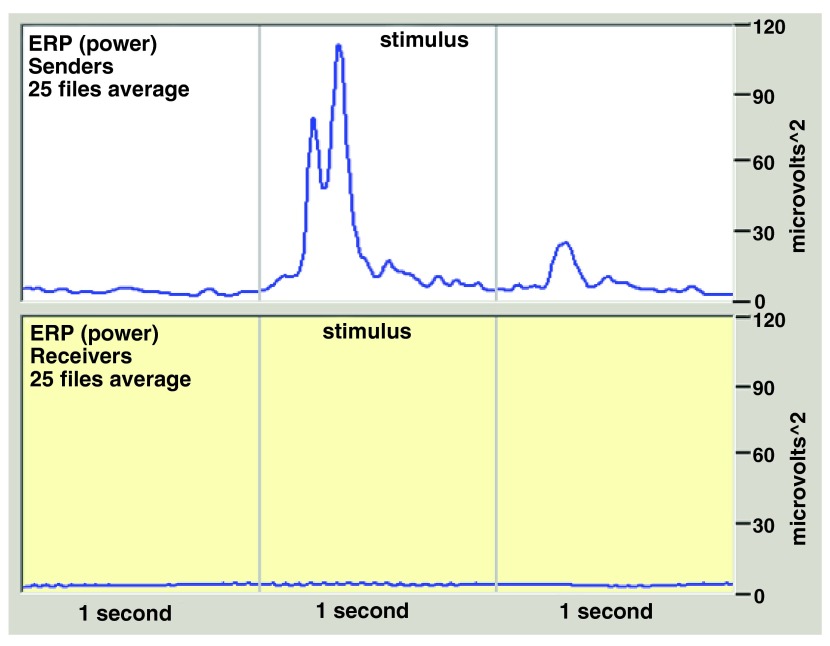
Results of overall average of ERP power: top graph is that of Senders, bottom shows that of Receivers.

The average Event-Related Potential (ERP) of all 25 files from Senders and Receivers was also calculated. To get the total sum of evoked potentials from all subjects while avoiding ERP different latency period problems, each subject’s individual evoked potential powers were added up. The resulting graph is shown in Figure 2.

Figure 2 clearly shows an ERP in the Senders, but nothing of interest in Receivers.

Following this negative result we began using an original method which we named GW6, created by one of our co-authors (WG), and described in detail in Tressoldi *et al.* (unpublished; pre-print proof available at http://biorxiv.org/content/early/2015/07/06/022046), which was found more resistant to jitter and interferences compared to the traditional averaging method. Furthermore, as described below and in more detail in the original paper, this new processing method is far less prone to unwanted effects of EEG artifact because as the Pearson Correlation depends only on signal phase and not to amplitude.

**Figure 3.**
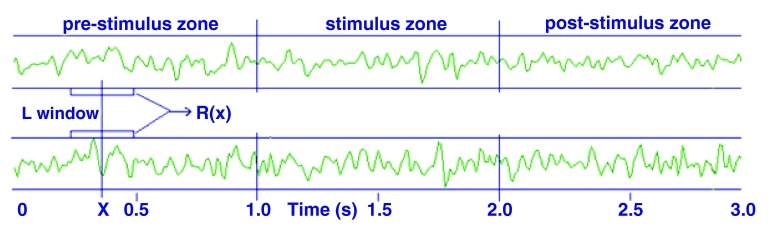
The window of length L runs along the traces left by two EEG channels. The corresponding Pearson Correlation is calculated and recorded on the R(x) array.

This method is based on the Pearson correlation between segments of data of fixed length L, as shown in Figure 3.

As an example, the Emotiv ^®^ EEG Neuroheadset provides NC = 14 EEG channels and a sample frequency of 128/s; the stimulus is 1 second duration and an epoch’s length is 3 seconds, equal to 384 samples. In this case it becomes possible to calculate the R(x) array in a number of combinations of pairs equal to: Nt = NC _*_(NC - 1)/2 = 91. The result can be written using a new array, R(I, X), in which I = 1... 91 and X = 1... 384 are the calculated values. The stimulus is administered at the same time as sample no. 128 and ceases after one second, with sample no. 256. The next processing step involves the average of R(I, X) over all the given stimuli, the result designated as R’(I, X).

Therefore, for each value of I = 1.... 91 a baseline is calculated, comprised of the average of R’(I, X) in the pre- and post- stimulus areas. Finally a new array is calculated, Sync(I, X), based on the result’s absolute value: Sync(I, X) = Abs(R’(I, X-Baseline)).

Then the average of all the Nt combinations gives us the final array, Sync1(X), which represents the total variations of the EEG correlations during a 3 second epoch, for all stimuli and all EEG channels. It is also possible to calculate a similar array, Sync2(C, X), for each channel, C.

**Figure 4.**
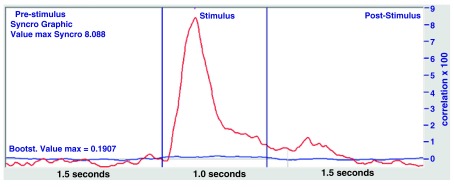
Overall result obtained from Senders and filtering all 25 EEG files from 1 to 16Hz + normalization, followed by application of the GW6 method. On the vertical axis are correlation values ×100. The blue curve denotes the average of 500 bootstrap files.

**Figure 5.**
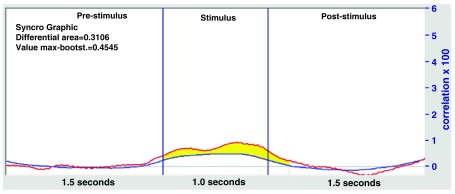
Overall result of 25 Receivers obtained from filtering the EEG signals in a narrow band, from 9 to 10 Hz + normalization, followed by application of GW6 method. The red curve is the average correlation and the blue is the bootstrap curve (average of 500 files), which represents the expected probability due to chance.

To be extra certain, the analysis of experimental data was nonetheless conducted on longer epochs – up to 4 seconds – comprised of 1.5 seconds pre-stimulus, 1 second stimulus, and 1.5 seconds post-stimulus. To calculate the probability that the observed differences in baselines are due to chance, the experimental data were compared to those obtained with a simulation conducted using a bootstrap procedure with the following characteristics:

a) Signals from the desired frequency range (in our case 9–10 Hz in Receivers and 1–16 Hz in Senders) are filtered using a digital filter that leaves signal phases intact, according to the Discrete Fourier Transform (DFT) and its related inverse processing. The filtered files are then saved. We point out that for Senders the standard frequency range (1–16 Hz) was used because the ERP is usually generated in this range.

b) The same processing method (The GW6 method) is applied to these files, but choosing at random the point in which a stimulus is thought to be present. For each file, the same number of stimuli (128) are evaluated, as in the experimental tests. For each file at least 20 bootstrap calculations are made, eventually resulting in over 500 files. The average of these calculations constitutes the blue bootstrap curve in Figure 4 and Figure 5, which therefore represents the expected probability due to chance, to compare with the obtained experimental curves (red). This method appears valid in that it gives a virtually flat curve (blue), close to zero throughout.

In Figure 4, a distinct peak (red curve) is seen which represents a correlation ERP at about 300 ms from the start of the stimulus, followed by a weaker peak at the end of the stimulus. This graph is similar to the one obtained from standard averaging in Figure 2, only expanded.

In Figure 5, with respect to the Receivers, there is an area that exceeds the normal chance expectation represented by the bootstrap curve. This area is highlighted in yellow and can be calculated as the difference with respect to the bootstrap curve.

A similar analysis was conducted by filtering signals within the 1 to 16 Hz band (as in the Senders) with statistically null results, and subsequently in the 8 to 16 Hz band, followed by the 8 to 12 Hz, but the best result was obtained in the 9 to 10 Hz range (see Table 1).

We concentrated our analyses on the alpha-theta bands which proven more sensible to distant correlations in previous similar studies.

**Table 1.** Area differences compared to bootstrap curves in three different signal filtration bands. The column on the right shows the probabilities that the results are purely due to chance.

Role	EEG Band	Area Difference	Maximum Value	Probability
**Receivers**	9–10	0.3106	0.4545	0.002/0.003
**Receivers**	8–12	0.1516	0.186	0.035/0.040
**Receivers**	8–16	0.0737	0.167	0.15/0.17
**Senders**	1–16	2.464	7.90	<0.00001

Due to the limitations of our EEG detection apparatus we did not proceed in a deep analysis of the sources (locations) of the observed effects, a very important detail. Preliminary analyses suggest the occipital and frontal locations as potential sources of the observed effects.

### Statistical control

After having established an increase in cerebral correlation in Receivers coinciding with the remote stimulus given to Senders, it is necessary to determine the importance of this difference with respect to the statistical chance. To this end, instead of resorting to conventional statistical methods, often inapplicable to complex situations such as this, we used an emulation procedure of the Monte Carlo type consisting of the following steps:

a) Take the bootstrap files within the desired frequency band – in our case from 9 to 10 Hz (around 500 files), or from other bands. All of these files are the result of a GW6-type processing and are now the input data set for the Monte Carlo emulation.

b) 25 “fake” files are randomly chosen and their final average is calculated, in the same way as the average of the 25 “real” files.

c) The difference in area with respect to the average of all the 500 bootstrap curves is calculated, as in Figure 5. This difference may be either a negative or positive number.

d) Determine if this number is higher than that obtained from the real experimental files. If it is higher, a counter is incremented.

e) Start again from a) and repeat the cycle as required (we repeated the cycle 2000 times).

**Figure 6.**
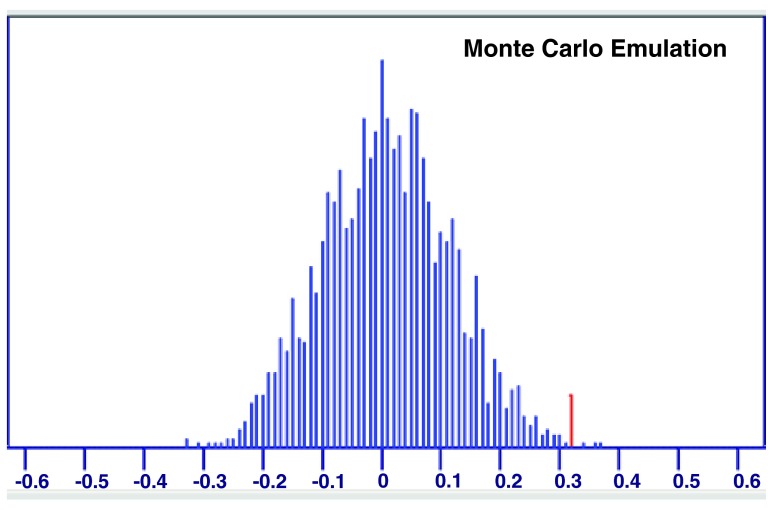
The graph shows the distribution of 2000 Monte Carlo emulations with respect to Receivers’ files filtered in the 9 to 10 Hz band. The value of the area to be exceeded (see Table 1) is 0.3106 (red line). The distribution is approximately Gaussian and the specified value is exceeded by chance 3 out of 2000 times.

At the end, determine how many times out of 1000 a group of 25 bootstrap files randomly exceeds the value of the area found experimentally. The results give the probability of obtaining a surplus of area by chance, as in Figure 6. This procedure does not use any specific a priori statistical model, and is based solely on applying numerous emulations exactly as with real data. As shown in Table 1, in the 9 to 10 Hz band the result is significant to a level of around 2–3/1000, equivalent to P ≤ 0.003, and is even significant in the 8 to 12Hz band, with P ≤ 0.04. The almost Gaussian distribution of the values shows that the method is valid and agrees with normal statistics.

## Discussion

When traditional methods of averaging and calculations for ERP power are used, there is a distinct evoked potential in the EEGs of subjects who are Senders, but not in the EEGs of Receivers.

Conversely, when the GW6 method is used and the signals within the 9–10 Hz band are filtered, we obtain the results shown in Figure 5, which are statistically confirmed by the Monte Carlo Emulation. These outcomes lead us to believe that Receivers exhibit a weak response to the remote stimulus in the form of a small change in cerebral synchronization coinciding with the stimulus. This variation approximately equates to a 0.5% correlation, with a maximum of about 1.5–2.0% in the best subjects under examination. Even though the applied method does not display a result in the form of a wave similar to that seen in the Senders’ ERPs, this result does however open the door to future investigations aimed at identifying specific patterns of weak but significant responses in Receivers. This study is clearly explorative but it is in agreement with the results observed in three different experiments by Hinterberger (2008) who observed an increase in the ERPs in the Alpha (8–12 Hz) band only in the related pairs of participants. If further confirmed, these findings would be of huge scientific importance because they provide neurophysiological evidence of a connection – or *social interaction* – at distance.

It is important to point out that our experimental design is by its nature not able to distinguish between classical and non-local interactions even if the GQT implies a “no-signal-transfer (NT) theorem” that is only an acausal correlation between two complex neurophysiological observables of two entangled subsystems of a total global system.

Regarding future developments in this area, we will attempt to identify EEG signals in Receivers while applying a gradual reduction in the number of stimulations. Continual advances in techniques for processing EEG signals allow us to be optimistic in reaching this objective.

## Data availability

The data referenced by this article are under copyright with the following copyright statement: Copyright: © 2016 Giroldini W et al.

The raw dataset and software codes for this article are available at: http://dx.doi.org/10.6084/m9.figshare.1466876

The unpublished proof describing the GW6 method is available at: http://biorxiv.org/content/early/2015/07/06/022046

## Figures and Tables

**Figure S1.**
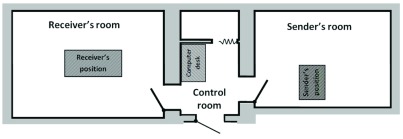
Floor plan showing rooms used in the experiment.

**Table S1.** Summary of the main characteristics of studies investigating Brain-to-Brain interaction at distance.

*Study*	*Year*	*n. pairs* *of data*	*Instruments*	*Stimulation*	*Dependent variables*
**Achterberger *et al.***	2005	10	fMRI	Distant Intention	F
**Ambach**	2008	17	EEG	Checkerboard patterns	F,C,P,O
**Burke, R. C., Gauthier, M. Y., Rouleau, N. &** **Persinger, M. A., Experimental Demonstration** **of Potential Entanglement of Brain Activity** **Over 300 Km for Pairs of Subjects Sharing the** **Same Circular Rotating, Angular Accelerating** **Magnetic Fields: Verification by s_LORETA,** **QEEG Measurements**	2013	2	EEG	Audio & Visual frequencies	Beta, **Alpha**, Theta
**Dotta *et al.***	2009	4	EEG	None	Theta, **Alpha**, Gamma
**Dotta *et al.***	2011	3	PMT	Magnetic fields + light flashes	Photons
**Duane, T.D., Behrendt, T., Extrasensory** **electroencephalographic induction between** **twins, Science 150 (1965) 367**	1965	15	EEG	Eye open-closed	**Alpha**
**Grinberg-Zylberbaum J, Delaflor M, Goswami A.** **The Einstein/Podolsky/Rosen paradox in the** **brain: The transferred potential.**	1994	1	EEG	Light flashes	VEPs
**Hearne**	1997	8	EEG	Stroboscope light	VEPs
**Hinterberger**	2010	86	EEG	Checkerboard pattern, IAPS	Theta, **Alpha**, Gamma
**Hearne**	1977	8	EEG	Light flashes	VEPs
**Hearne**	1981	11	EEG	Tachistoscopic light	VEPs
**Kittenis**	2004	18	EEG	Single flashes	**Alpha**
**Manolea**	2015	16	EEG	IAPS	Theta
**Millar**	1975	20	EEG	Light flashes	VEPs
**Orme-Johnson *et al.***	1982	3	EEG	Transcendental Meditation	**Alpha**, Beta
**Persinger *et al.***	2008	4	EEG	Magnetic fields + light flashes	Theta, **Alpha**
**Persinger *et al.***	2010	10	EEG	Magnetic fields + light flashes	**Alpha**
**Persinger *et al.***	2003	4	EEG	Magnetic fields	Theta, **Alpha**
**Radin**	2004	13	EEG	Image	Cz ERP
**Rebert & Turner**	1974	1	EEG	Light flashes	**Alpha**
**Richards *et al.***	2005	1	fMRI -EEG	Checkerboard patterns	O, **Alpha**
**Scott *et al.***	2015	5	EEG	light visualization	Theta, Gamma
**Standish *et al.***	2003	1	fMRI	Checkerboard patterns	O
**Standish *et al.***	2004	60	EEG	Checkerboard patterns	O, VEPs
**Targ & Puthoff**	1974	6	EEG	Light flashes	**Alpha**
**Tressoldi *et al.***	2014	20	EEG	Image + sound	**AF3, F7, F3, FC5, T7,** **P7, O1, O2, P8, T8,** **FC6, F4, F8, AF4**
**Ventura *et al.***	2014	8	EEG	Reiki	Theta, **Alpha**, Gamma
**Wackermann *et al.***	2003	17	EEG	Checkerboard patterns	VEPs
**Wackermann *et al.***	2004	16	EEG	Checkerboard patterns	VEPs

## Supplementary Material

**Studies investigating Brain-to-Brain interaction at distance**

Achterberg, J., Cooke, K., Richards, T., Standish, L. J., Kozak, L., & Lake, J. (2005). Evidence for correlations between distant intentionality and brain function in recipients: A functional magnetic resonance imaging analysis. *Journal of Alternative & Complementary Medicine: Research on Paradigm, Practice, and Policy*, 11(6), 965–971.

Ambach, W. (2008). Correlations between the EEGs of two spatially separated subjects—a replication study. *European Journal of Parapsychology*, 23,2, 131–146. http://ejp.wyrdwise.com/EJP v23-2.pdf

Burke, R. C., Gauthier, M. Y., Rouleau, N. & Persinger, M. A. (2013). Experimental Demonstration of Potential Entanglement of Brain Activity Over 300 Km for Pairs of Subjects Sharing the Same Circular Rotating, Angular Accelerating Magnetic Fields: Verification by s_LORETA, QEEG Measurements. *Journal of Consciousness Exploration & Research*, 4, 1, 35–44.

Dotta, B. T., Mulligan, B. P., Hunter, M. D., & Persinger, M. A. (2009). Evidence of macroscopic quantum entanglement during double quantitative electroencephalographic measurements of friends vs strangers. *NeuroQuantology*, 7(4), 548–551.

Dotta, B. T., Buckner, C. A., Lafrenie, R. M., & Persinger, M. A. (2011). Photon emissions from human brain and cell culture exposed to distally rotating magnetic fields shared by separate light-stimulated brains and cells. *Brain research*, 1388, 77–88.

Duane, T. D., & Behrendt, T. (1965). Extrasensory electroencephalographic induction between identical twins. *Science*, 367.

Grinberg-Zylberbaum, J., Delaflor, M., Attie, L., and Goswami, A. (1994). The Einstein-Podolsky-Rosen Paradox in the brain: the transferred potential. *Physics Essays*, 7, 422–427.

Hearne K. (1977). Visually evoked responses and ESP. *Journal of the Society of Psychic Research*, 49: 648–657.

Hearne, K. (1981). Visually evoked responses and ESP: Failure to replicate previous findings. *Journal of the Society of Psychic Research*, 51: 145–147.

Hinterberger, T. (2008). Searching for Neuronal Markers of Psi: A Summary of Three Studies Measuring Electrophysiology in Distant Participants”, in Utrecht II: Charting the Future of Parapsychology, Proceedings of an International Conference held in Utrecht, 2008, Parapsychology Foundation, NY, pp. 46–62. ISBN: 978–1–931747–28–8.

Kittenis M, Caryl P, Stevens P. (2004). Distant psychophysiological interaction effects between related and unrelated participants, Proceedings of the Parapsychological Association Convention, Vienna, Austria, August 5–8, 67–76.

Manolea, A. (2015). Brain to Brain Connectivity During Distal Psycho-informational Influence Sessions, Between Spatially and Sensory Isolated Subjects. *Procedia-Social and Behavioral Sciences*, 187, 250–255.

Millar, B. (1975). An attempted validation of the “Lloyd effect.”. *Research in Parapsychology*, 25–27.

Orme-Johnson, D., Dillbeck, M. C., Wallace, R. K., & Landrith, G. S. (1982). Intersubject EEG coherence: Is consciousness a field? *International Journal of Neuroscience*, 16(3–4), 203–209.

Persinger, M.A. Tsang, E.W. Booth, J.N. Koren, S.A. (2008). Enhanced power within a predicted narrow band of theta activity during stimulation of another by circumcerebral weak magnetic ﬁelds after weekly spatial proximity: evidence for macroscopic entanglement? *Neuroquantology,* 6, 7–21.

Persinger, M. A., Saroka, K. S., Lavallee, C. F., Booth, J. N., Hunter, M. D., Mulligan, B. P.,... & Gang, N. (2010). Correlated cerebral events between physically and sensory isolated pairs of subjects exposed to yoked circumcerebral magnetic fields. *Neuroscience Letters*, 486(3), 231–234.

Persinger, M. A., Koren, S. A., & Tsang, E. W. (2003). Enhanced power within a specific band of theta activity in one person while another receives circumcerebral pulsed magnetic fields: a mechanism for cognitive influence at a distance? *Perceptual and Motor Skills*, 97(3), 877–894.

Puthoff, H., & Targ, R. (1974). Information transmission under conditions of sensory shielding. *Nature*, 252(5476), 602–607.

Radin, D. I., (2004). Event-related electroencephalographic correlations between isolated human subjects. *The Journal of Alternative and Complementary Medicine*, 10, 315–323.

Rebert, C. S., & Turner, A. (1973). EEG spectrum analysis techniques applied to the problem of psi phenomena. *Behavioral Neuropsychiatry*, 6(1–12), 18–24.

Richards, T. L., Kozak, L., Johnson, L. C., & Standish, L. J. (2005). Replicable functional magnetic resonance imaging evidence of correlated brain signals between physically and sensory isolated subjects. *Journal of Alternative & Complementary Medicine: Research on Paradigm, Practice, and Policy*, 11(6), 955–963.

Scott, M. A., Rouleau, N., Lehman, B. S., Tessaro, W. E., Juden-Kelly, L. M., Saroka, K. S. & Persinger, M. A., (2015). Experimental Production of Excess Correlation across the Atlantic Ocean of Right Hemispheric Theta-Gamma Power between Subject Pairs Sharing Circumcerebral Rotating Magnetic Fields (Part I, Part II). *Journal of Consciousness Exploration & Research*, 6, 9, 658–684, 685–707.

Standish, L. J., Johnson, L. C., Kozak, L., & Richards, T. (2003). Evidence of correlated functional magnetic resonance imaging signals between distant human brains. *Alternative Therapies in Health and Medicine*, 9(1), 128–128.

Standish, L. J., Kozak, L., Johnson, L. C., and Richards, T. (2004). Electroencephalographic evidence of correlated event-related signals between the brains of spatially and sensory isolated human subjects. *The Journal of Alternative and Complementary Medicine*, 10, 307–314.

Tressoldi P, Pederzoli L, Bilucaglia M *et al.* (2014). Brain-to-Brain (mind-to-mind) interaction at distance: a confirmatory study [v3; ref status: approved 1, not approved 1, http://f1000r.es/4ka] *F1000Research*, 3:182 (doi: 10.12688/f1000research.4336.3).

Ventura, A. C., Saroka, K. S., & Persinger, M. A. (2014). Non-Locality changes in intercerebral theta band coherence between practitioners and subjects during distant Reiki procedures. *Journal of Nonlocality*, 3(1), 1–25.

Wackermann, J., Seiter, C., Keibel, H., and Walach, H. (2003). Correlations between brain electrical activities of two spatially separated human subjects. *Neuroscience Letters*, 336, 60–64.

Wackermann, J., Naranjo Muradás, J.R., and Pütz, P. (2004). Event-Related Correlations between Brain Electrical Activities of Separated Human Subjects: Preliminary Results of a Replication Study. *Proceedings of the 47th Annual Convention of the Parapsychological Association*, 465–468.
