# Supplementary material for: EEG correlates of social interaction at distance
Source: F1000Res. 2016 Jan 14;4:457. Originally published 2015 Aug 3. [Version 3] doi: 10.12688/f1000research.6755.3 (PMC4770988; doi:10.12688/f1000research.6755.3)
Supplement: Peer review history

# Peer review: Referee response for version 3

**DOI:** 10.5256/f1000research.8396.r11137

**Published:** 2016-01-22

**Authors:** Thilo Hinterberger

**Affiliations:** 1 Research Section of Applied Consciousness Sciences, Department of Psychosomatic Medicine, Medical Center, University of Regensburg, Regensburg, Germany

1. In the Introduction you write:
  “Our study is a further contribution to this line of research, but for the first time within the social neuroscience and the GQT framework.”
  I do not understand why you think that you for the first time link GQT and social neuroscience. You only did an experiment of the type many others did before in a very similar way. Therefore, please omit the second half of the sentence.
2. I do not understand the meaning of this sentence and the NT seems to be strangely introduced here:
  “It is important to point out that our experimental design is by its nature not able to distinguish between classical and non-local interactions even if the GQT implies a “no-signal-transfer (NT) theorem” that is only an acausal correlation between two complex neurophysiological observables of two entangled subsystems of a total global system.”
  My suggestion to indicate the limitations with respect to the GQT is:
  “It is important to point out that our experimental design is by its nature not able to distinguish between classical and non-local interactions. As in quantum physics, also here a Bell-type experiment would be necessary to distinguish between classical signal transfer and nonlocal correlation. Instead, here we only looked for correlations similarly to other studies of this type (see References)."

I have read this submission. I believe that I have an appropriate level of expertise to confirm that it is of an acceptable scientific standard, however I have significant reservations, as outlined above.

# Response

**Published:** 2016-01-22

**Authors:** Patrizio Tressoldi

**Affiliations:** Dipartimento di Psicologia Generale, Università di Padova, Italy

In the new version we updated the introduction and the discussion following your suggestions

# Peer review: Referee response for version 2

**DOI:** 10.5256/f1000research.7913.r11391

**Published:** 2015-12-01

**Authors:** Thilo Hinterberger

**Affiliations:** 1 Research Section of Applied Consciousness Sciences, Department of Psychosomatic Medicine, Medical Center, University of Regensburg, Regensburg, Germany

Thank you for the improvements and adding the list of studies. I still have some concerns which I think should be regarded in order to be scientifically correct. They refer to your responses on my points raised above:

2. I still do not see all preconditions for the applicability of the GQT fulfilled. As you do not show that you should not state that "this relationship constitutes an entangled state". The GQT cannot be applied to any complex system. There are complex systems that behave just classically when split up into parts. Further, the random generator experiment of Walach can be connected with GQT as it analyses the matrix of physical and psychological variables and thereby excludes a signal transfer. In this aspect, your experimental design is rather of a classical nature and its non-local entanglement nature cannot be proven with it. Therefore, again, I suggest you to be more careful in the introduction.

3. Again, why did you choose the bands 1-16, 8-16, 8-12 and 9-10Hz? Why not the standard EEG bands? Or have you analyzed the other frequency bands? Then, it would be honest to report also the other frequency bands and correct for multiple testing. Searching for the highest effect in the data and reporting only this is statistically invalid.

6. If possible, please provide a mapping or some information showing the spatial distribution of the effect. Should be no problem as you have the data and the algorithms.

I have read this submission. I believe that I have an appropriate level of expertise to confirm that it is of an acceptable scientific standard, however I have significant reservations, as outlined above.

# Response

**Published:** 2015-12-05

**Authors:** Patrizio Tressoldi

**Affiliations:** Dipartimento di Psicologia Generale, Università di Padova, Italy

2. I still do not see all preconditions for the applicability of the GQT fulfilled. As you do not show that you should not state that "this relationship constitutes an entangled state". The GQT cannot be applied to any complex system. There are complex systems that behave just classically when split up into parts. Further, the random generator experiment of Walach can be connected with GQT as it analyses the matrix of physical and psychological variables and thereby excludes a signal transfer. In this aspect, your experimental design is rather of a classical nature and its non-local entanglement nature cannot be proven with it. Therefore, again, I suggest you to be more careful in the introduction.

**Reply: It seems your concerns are related to our statements:**

**“ *A distant social interaction between two persons who know each other satisfies these requirements [for mind-to-mind and mind-to-matter non-local correlations] provided that: ***

- ***a) the two persons represent two subsystems of a single larger one created by their relationship, and***
- ***b) this relationship constitutes an entangled state, and furthermore that***
- ***c) the measurable psychological and physiological variables represent the system’s comprehensive characteristic even though measured individually*** **.”**

**We agree that these hypotheses must be tested empirically, but are coherent with those put forward by Walach *et al. *(in press) in testing mind-to-matter non local correlations postulating an entanglement between participants and the RNG.**

**What if in the new version we introduce the above paragraph with the following premise: “ *When dealing with mental observables, the identification and operationalization of the subsystems within a global one, and their complementary and/or compatible characteristics is still an open problem. In the present study we will assume that…*” ?**

**As far as the no-signal-transfer (NT) theorem, in the third paragraph of the Discussion section of version 2 we explicitly declared that “… *what we are observing is not a transmission of signals from the sender to receiver participants, but only a correlation between two complex neurophysiological observables*..”**

**3. **Again, why did you choose the bands 1-16, 8-16, 8-12 and 9-10Hz? Why not the standard EEG bands? Or have you analyzed the other frequency bands? Then, it would be honest to report also the other frequency bands and correct for multiple testing. Searching for the highest effect in the data and reporting only this is statistically invalid.

**Reply: We agree. In the new version we will explicitly declare that we concentrated our analyses on the alpha-theta bands which in previous similar studies proven more sensible to nonlocal correlations.**

6. If possible, please provide a mapping or some information showing the spatial distribution of the effect. Should be no problem as you have the data and the algorithms.

**Reply: We agree that this information could add more details to the characteristics of the nonlocal correlations, but given the limitations of our EEG apparatus, we did not proceed at present time in the source analysis.**

**In the future studies we will add the search of the spatial  distribution of the effect and the  more active locations.**

**In the new version we will add this clarification.**

**Please let us know whether you agree with these revisions before we submit version 3.**

# Response

**Published:** 2016-01-11

**Authors:** Thilo Hinterberger

**Affiliations:** University Clinic of Regensburg, Germany

Regarding 2:

I would suggest instead of: "A distant social interaction between two persons who know each other satisfies these requirements..."to write: "The theory of Generalized Entanglement assumes, that a distant social interaction between two persons who know each other must satisfy these requirements..."

Then, as you suggested please insert the sentence:

“When dealing with mental observables, the identification and operationalization of the subsystems within a global one, and their complementary and/or compatible characteristics is still an open problem. In the present study we will assume that…”

Regarding my comment on non-signal transfer:

My point of critics was not that you expect "only" correlations in your experiment. It is more that you should state that your experimental design is by its nature not able to distinguish between classical and non-local interactions because if your experiment is successful and replicable, it can be used for signal transfer as well. You should add a sentence which makes this clear. Therefore, your sentence "…what we are observing is not a transmission of signals from the sender to receiver participants, but only a correlation..." is wrong because you don't prove that. These kind of correlations still could have a classical explanation!

Regarding 3:

OK. But please don't say " ...more sensible to nonlocal correlations." Better would be " ...more sensible to distant correlations." as I said, you don't prove non-locality.

Regarding 6:

Some hints about the contribution of electrode positions to the effect can be given without calculating source localizations. You can keep this simple.

# Response

**Published:** 2016-01-12

**Authors:** Patrizio Tressoldi

**Affiliations:** Dipartimento di Psicologia Generale, Università di Padova, Italy

Thank you very much for these clarifications we have now considered in the new version of the paper. On pag. 1 we have clarified which requirements are necessary to satisfy the condition for a Generalized Entanglement between two distant persons. On pag. 8 we clarified why we did not proceed in the source analysis and on pag. 9, how our findings fit with the  no-signal-transfer (NT) theorem.

# Peer review: Referee response for version 1

**DOI:** 10.5256/f1000research.7257.r10191

**Published:** 2015-09-29

**Authors:** Aliodor Manolea

**Affiliations:** 1 Faculty of Psychology and Education Sciences, University of Bucharest, Bucharest, Romania

1. The work is interesting and well done, in terms of the experimental work and data processing, both part of EEG signal processing and a statistical approach.
2. The abstract describes very precisely the content of the article. The addressed  research stage is clearly exposed and has direct addressability with the experiment described in this article and it is well supported by bibliographic references.
3. The analysis method of ERP (The GW6 method), used to extract useful information from the noise, seemed to me very appropriate in the context of the uncertainty of ERP occurrence in the EEG recordings  of the receivers.
4. Also, statistical analysis is well designed and properly made, it allowed the exclusion of chance from investigated phenomenology.
5. The effect size in such experiments is small or very small, being on the border of chance. Therefore, it would have been appropriate to make a comparison with the effect size obtained in other experiments of this type.
6. The results are well supported by the experimental data and the processing methods. I think  that the authors provide sufficient data to replicate the experiment without having great difficulties, which supports the scientific nature of the research.
7. Also, I say that I agree with changes made at the suggestion of one of the referees.
8. For future research I think that generating a baseline obtained from experimental sessions in which any (no) stimulus will be applied, It would be more appropriate than the method used in this experiment.

I have read this submission. I believe that I have an appropriate level of expertise to confirm that it is of an acceptable scientific standard.

# Response

**Published:** 2015-10-31

**Authors:** Patrizio Tressoldi

**Affiliations:** Dipartimento di Psicologia Generale, Università di Padova, Italy

Thank you for your review. Here follows our replies to your comments.

......

5. The effect size in such experiments is small or very small, being on the border of chance. Therefore, it would have been appropriate to make a comparison with the effect size obtained in other experiments of this type.

**Reply: We are still wondering if a meta-analysis could be carried out on all the available studies related to this phenomena, but the variety of methods used to analyse the data (see the Supplementary Material), raise serious concerns on this possibility.**

.....

8. For future research I think that generating a baseline obtained from experimental sessions in which any (no) stimulus will be applied, It would be more appropriate than the method used in this experiment.

**Reply: We agree with your suggestion we will take it in account for the next experiments.**

# Peer review: Referee response for version 1

**DOI:** 10.5256/f1000research.7257.r9814

**Published:** 2015-09-10

**Authors:** Thilo Hinterberger

**Affiliations:** 1 Research Section of Applied Consciousness Sciences, Department of Psychosomatic Medicine, Medical Center, University of Regensburg, Regensburg, Germany

The manuscript reports a study in which spatially separated pairs of participants have been measured with EEG simultaneously. One of them was audiovisually stimulated at random times. The hypothesis was tested whether an evoked response related to the stimulation in one participant could be detected in the EEG of the non-stimulated participant. A nonparametrical analysis using bootstrapping analysis showed a positive effect in the alpha frequency range.

The manuscript is written clearly and the experiment and analysis seem to be done in a scientific rigor way. However, several questions arose and some additions and changes should be done before full approval of this paper. These are described in the following:

1. This type of experiment was already done and published by various researchers. Almost none of them is mentioned but I think this is important as the study is a kind of replication of previous studies. Especially, as there are experiments that showed an effect in the alpha band which supports your findings. I would suggest to report previous studies of this type in the introduction and shortly report their findings (E.g.by  Wackermann, Radin, Hinterberger,…).
2. In the introduction the Generalized Quantum Theory is presented as a rational for the hypothesis. I would be very careful with this approach because it is not clear whether this study design can be used to test the WQT and further whether the WQT actually applies to the phenomenon of distant mental interactions even if it is an elegant model. Spoken as a physicist, I do not really see the conditions for entanglement fulfilled because not every subsystem, that has information of another subsystem and shares the idea of interconnectedness behaves as being entangled. Therefore, it seems to be oversimplified to just state that "this relationship constitutes an entangled state".
  Another problem arises with the non-signal transfer paradigm which in this type of experiment would be violated if we would find replicable correlations of the same type. In such case one should find a different explanation for the effect.
  Therefore, I suggest instead of claiming the WQT in the introduction, the study should be presented as a replication of previous studies in a slightly different manner, testing an experiential phenomenon reported by many people. Also please state why you were using this type of stimuli. The WQT would probably fit better as an attempt for an explanation of the findings in the discussion or, if used in the introduction with much more care.
3. Why were you focusing on the frequency range between 9-10 Hz? Was this a post-hoc selection? Could you tell the results of the other standard frequencies such as theta, beta,.. bands?
4. “…it becomes possible to calculate the R(x) array in a number of combinations of pairs equal to: Nt = NC _*_(NC - 1)/2 = 91. The result can be written using a new array, R(I, X), in which I = 1... 91 and X = 1... 384 are the calculated values.“
  This description remains unclear to me. Could you describe the meaning of the variables and explain the process a bit more?
5. What is the GW6 method?
6. Is it possible to tell something about the localization of your findings as you recorded 14 channels?
7. In the discussion please compare your findings to those of similar studies.

I have read this submission. I believe that I have an appropriate level of expertise to confirm that it is of an acceptable scientific standard, however I have significant reservations, as outlined above.

# Response

**Published:** 2015-10-31

**Authors:** Patrizio Tressoldi

**Affiliations:** Dipartimento di Psicologia Generale, Università di Padova, Italy

Thank you for your review. Here follows our replies to your comments.

1. ......I would suggest to report previous studies of this type in the introduction and shortly report their findings (E.g. by Wackermann, Radin, Hinterberger,…).

**Reply: In the introduction and in the discussion of version 2, we added this information. Furthermore we added the list and references of all studies related to this line of investigation in the Supplementary Material.**

2 **. ....**Spoken as a physicist, I do not really see the conditions for entanglement fulfilled because not every subsystem, that has information of another subsystem and shares the idea of interconnectedness behaves as being entangled. Therefore, it seems to be oversimplified to just state that "this relationship constitutes an entangled state" **.**

**Reply: We are aware of your knowledge regarding the theory, and as explained by the GQT authors, this theory is not related to physical observables, but rather is one that can be applied to every complex system, regardless of whether physical, biological, or mental. If we assume that every human mind is a complex system, it is safe to assume the possibility that two minds, a certain distance apart, can be entangled if the intention to be connected is mutual, as suggested in the instructions to participants. This theory has already obtained empirical support in the entanglement of a human mind with a random number generator – considered to be a complex physical system (Walach *et al*. PLoS *in press*).**

...Another problem arises with the non-signal transfer paradigm which in this type of experiment would be violated if we would find replicable correlations of the same type.

**Reply: We agree. In the Discussion we clarify that what we observed is a correlation and not a transmission of signals.**

**3.** Why were you focusing on the frequency range between 9-10 Hz? Was this a *post-hoc *selection? Could you tell the results of the other standard frequencies such as theta, beta,.. bands?

**Reply: In the Discussion we** **specifically stated the explorative nature of the study. No other relevant results were observed in other EEG frequency bands.**

**4-5. **What is the GW6 method?

**Reply: As explained in the Data Analysis section, this is an alternative method for analyzing EEG signals based on the correlation among the signals detected in the EEG channels (14 in our case), which is more resistant to jitter and interferences compared to simple classical ERP averaging. The cited and freely available reference (Giroldini *et al*) contains a more technical explanation of how it works and how it differs from classical methods.**

**6. **Is it possible to tell something about the localization of your findings as you recorded 14 channels?

**Reply: We did not investigate the “sources” of the observed correlation given that our primary interest was in extracting it from the noise present in the EEG signals. We agree that a comparative analysis of the sources of the correlation, for example frontal versus occipital between the senders and the receivers, may give important clues about its characteristics.**

**7. **In the discussion please compare your findings to those of similar studies.

**Reply: Added.**
